# The rewiring of cAMP/cGMP and LDH signalling drives cardiac hypertrophy in *Pde5a*^*−/−*^ mice

**DOI:** 10.26508/lsa.202403094

**Published:** 2025-07-14

**Authors:** Ana Gabriela de Oliveira do Rêgo, Sonia Maccari, Giuseppe Marano, Tonino Stati, Michele Saliola, Mauro Giorgi, Silvia Cardarelli, Matilde Merolle, Francesco Tomassoni-Ardori, Lino Tessarollo, Federica Campolo, Andrea M Isidori, Luciana De Angelis, Fabio Naro, Lucia Monaco, Manuela Pellegrini

**Affiliations:** 1 Department of Anatomical, Histological, Forensic and Orthopaedic Sciences, Sapienza University of Rome , Rome, Italy; 2 Center for Gender Medicine, Istituto Superiore di Sanità , Rome, Italy; 3 Department of Biology and Biotechnology “C. Darwin”, Sapienza University , Rome, Italy; 4 Neural Development Section, Mouse Cancer Genetics Program, Center for Cancer Research, NCI, NIH, Frederick, MD, USA; 5 Department of Experimental Medicine, Sapienza University , Rome, Italy; 6 Department of Physiology and Pharmacology, Sapienza University , Rome, Italy; 7 Institute of Biochemistry and Cell Biology, IBBC-CNR, Rome, Italy

## Abstract

Phosphodiesterase type 5a (Pde5a), an enzyme that hydrolyzes cGMP nucleotide, regulates several aspects of heart physiology and its inhibition improves left ventricular heart function under pathological conditions. We investigated Pde5a role in the development and progression of moderate and severe cardiac hypertrophy, induced by transverse aortic constriction (TAC), in Pde5a WT (*Pde5a*^*+/+*^) and Pde5a-deficient (*Pde5a*^*−/−*^) mice. Cardiac hypertrophy was surprisingly detected in *Pde5a*^*−/−*^ mice after TAC surgery with similar alterations of cardiac function, morphology, fibrosis, and expression of molecular markers compared with *Pde5a*^*+/+*^ mice. The Pde5a inhibitor, Sildenafil, prevented only moderate cardiac hypertrophy at the morpho-functional and molecular levels in *Pde5a*^*+/+*^ mice. Cardiac hypertrophy was found to be associated with unbalanced cAMP/cGMP ratio and lactate dehydrogenase plays a critical role in the metabolic remodelling of *Pde5a*^*−/−*^ hearts under TAC conditions. In conclusion, pharmacological Pde5a inhibition counteracts moderate but not severe hypertrophy. Genetic ablation of Pde5a does not protect against moderate/severe cardiac hypertrophy. In the absence of Pde5a, the oxidative metabolism shifts towards a mixed oxidative-glycolytic metabolism under TAC condition.

## Abstract (precis)

This discovery will be very useful in term of personalized medicine because Pde5a inhibitors should be administrated depending on the severity of cardiac hypertrophy. Moreover, the Pde5a/Sildenafil system counteracts a metabolic rewiring occurring in cardiac hypertrophy.

## Introduction

Phosphodiesterases (Pdes) are components of several cellular processes through their ability to modulate the second messenger signalling molecules, cAMP and cGMP (1, 2). In addition, their localization in different subcellular compartments also contributes to the spatial and temporal distribution of cyclic nucleotide pools (3, 4, 5). Pdes are responsible for the maintenance of cyclic nucleotides homeostasis, and their alteration contributes to the induction/progression of pathological conditions in several systems (6, 7, 8, 9, 10, 11).

Among the cGMP-Pdes, Pde5a was reported to be expressed in the heart since the first stages of murine embryogenesis (12) and three isoforms (Pde5a1, a2, and a3) generated from alternative splicing showed different levels of expression and subcellular localization in cardiomyocytes (13). Under physiological conditions, Pde5a is present at a low level in cardiomyocytes, and its localization is restricted to the Z-disc (14, 15). Increased expression/activity of Pde5a was detected in cardiovascular alterations of human patients and in animal models of cardiac diseases (16, 17, 18, 19). Several clinical trials have been performed or are still ongoing for the use of Pde5a inhibitors in different cardiovascular disorders (www.clinicaltrials.gov). Results showed that, in most but not all cases, chronic pharmacological inhibition of Pde5a is correlated with improved hemodynamic parameters and cardiac performance (7, 9, 20, 21). Cardiac alterations were also attenuated by pharmacological inhibition of Pde5a activity in some animal models (22, 23, 24, 25). It was reported that the Pde5a inhibitor Sildenafil had cardioprotective effects, through protein kinase G (PKG) activation, in mouse heart hypertrophy, mainly in severe hypertrophy, that was induced by transverse aortic constriction (TAC) (22, 26, 27, 28). On the contrary, by using mice lacking the beta isoform of the cGMP-kinase type I (cGKI/PKGI) in cardiomyocytes, it was described that this kinase is not involved in the development of heart hypertrophy after TAC, suggesting that the modulation of cardiac hypertrophy can occur not exclusively through the Pde5a-cGMP-PKG pathway (29).

Overall, it has been proposed that Pde5a inhibitors may exert their cardiac protective role by either interfering with PKG function (10, 30) or blocking/decreasing inflammatory response (31, 32) or mediating the action of the mitochondrial regulator PGC1α (33), but the exact mechanisms underlining the beneficial effects of Pde5a inhibition in cardiovascular pathologies still need to be clarified.

To better investigate this aspect and to clarify the role of Pde5a in cardiac hypertrophy induced by TAC, we used, for the first time, genetically modified mice deficient in Pde5a (*Pde5a*^*−/−*^) and we compared the responses with experimentally induced hypertrophy with or without Sildenafil. The results revealed differences between pharmacological inhibition and genetic ablation targeting Pde5a in induced cardiac hypertrophy. Moreover, we found peculiar metabolic changes triggered by Pde5a inhibition in adaptive cardiomyocytes after hypertrophy.

## Results

### Basal and isoproterenol-stimulated cardiac function is preserved in *Pde5a*^*−/−*^ mice

**Figure 1.**
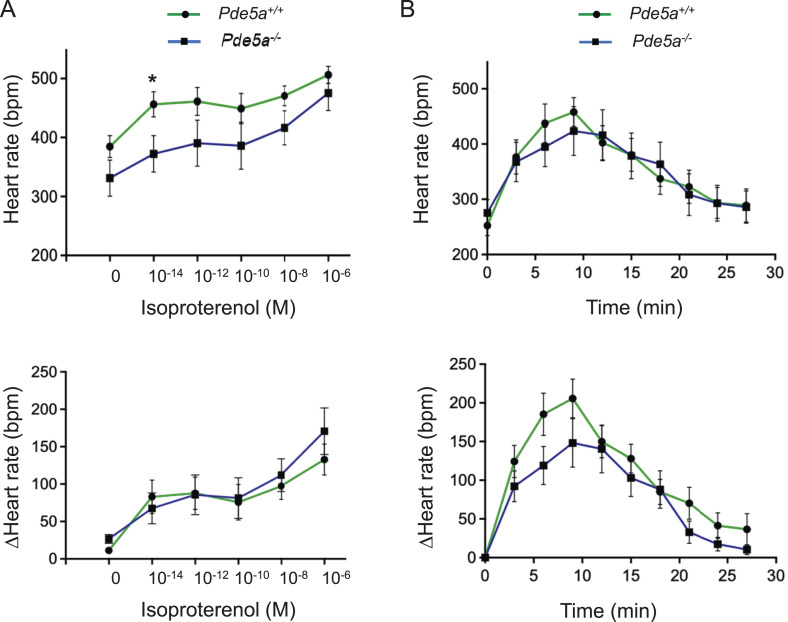
Beating rates in hearts isolated from *Pde5a*^*+/+*^ and *Pde5a*^*−/−*^ mice in basal condition and after beta-adrenergic stimulation. **(A)** Heart rate response to perfusion with different concentrations of isoproterenol was measured as beats per minute (bpm) by a Langendorff system (upper panel); Δ hearts rate: beats while perfusing with isoproterenol subtracted by resting cardiac rate (lower panel). **(B)** Heart rate taken at various times in isolated hearts receiving single pulse of 10^−7^ M isoproterenol (upper panel). Δ hearts rate: beats while perfusing with isoproterenol subtracted by resting cardiac rate (lower panel). For each experiment, eight mice for a group were analysed and one-way ANOVA was performed (*P ≤ 0.05).

Initially, the Langendorff system was used in ex vivo isolated hearts to understand the cardiac physiology and evaluate myocardial effects on the coronary circulation of Pde5a^*−/−*^ mice. Basal heart rate was similar between *Pde5a*^*−/−*^ mice compared with *Pde5a*^*+/+*^ (Fig 1A). Then adrenergic stimulation was tested by heart perfusion with different doses of isoproterenol, a beta-adrenergic agonist (10^−14^–10^−6^ M). *Pde5a*^*−/−*^ hearts could respond to isoproterenol stimulation even with a slightly lower rate than WT hearts (Fig 1A, upper panel). Differences entirely disappeared when delta heart rate was considered (Δ Heart rate) (Fig 1A, lower panel). Hearts were then perfused with a constant dose of isoproterenol (10^−7^ M) for 30 min and frequency was recorded every 3 min (Fig 1B). No significant differences in either the peak of stimulation or the return to baseline phases were observed in the two genotypes (Fig 1B, upper panel). A slight trend of decrease in the peak was detected when delta beats were measured (Fig 1B, lower panel). These results suggest that *Pde5a*^*−/−*^ hearts show normal heart rates under basal and acute β-adrenergic stimulation.

### Cardiac functions and morphology are not preserved in *Pde5a*^*−/−*^ mice after moderate TAC-induced cardiac hypertrophy but only after Pde5a inhibition

To evaluate the role of Pde5a in cardiac hypertrophy, the TAC protocol, previously described (22), was used in *Pde5a*^*+/+*^ and *Pde5a*^*−/−*^ 3-mo-old mice. Moderate and severe cardiac hypertrophy was produced by narrowing the aortic arch, using needles of 26G or 27G thickness, respectively. Both *Pde5a*^*+/+*^ and *Pde5a*^*−/−*^ male mice underwent surgery, and one group of each concomitantly received the Pde5a inhibitor Sildenafil citrate (SILD). Mice were divided into three groups named SHAM, TAC, and TAC+SILD. The phenotype was evaluated 4 wk after surgery, monitoring the cardiac function by echocardiogram, the morphology by histological analyses, and subsequently analysing the main molecular mechanisms involved in cardiac hypertrophy. The parameters used to distinguish between hypertrophied and non-hypertrophied hearts included cardiac mass, cardiomyocyte size, fractional shortening, ejection fraction, and blood flow velocity, as detailed in Table S1.

Table S1. Cutoff of parameters used to select mice with heart hypertrophy.

Heart rate, left ventricular diameter, and left ventricular wall thickness in systole and diastole were evaluated by echocardiography in *Pde5a*^*+/+*^ and *Pde5a*^*−/−*^ experimental groups. Except for the left ventricular diameter, these parameters did not change significantly after TAC or after treatment with Sildenafil (Table S2A).

Table S2. Echo parameters.

**Figure 2.**
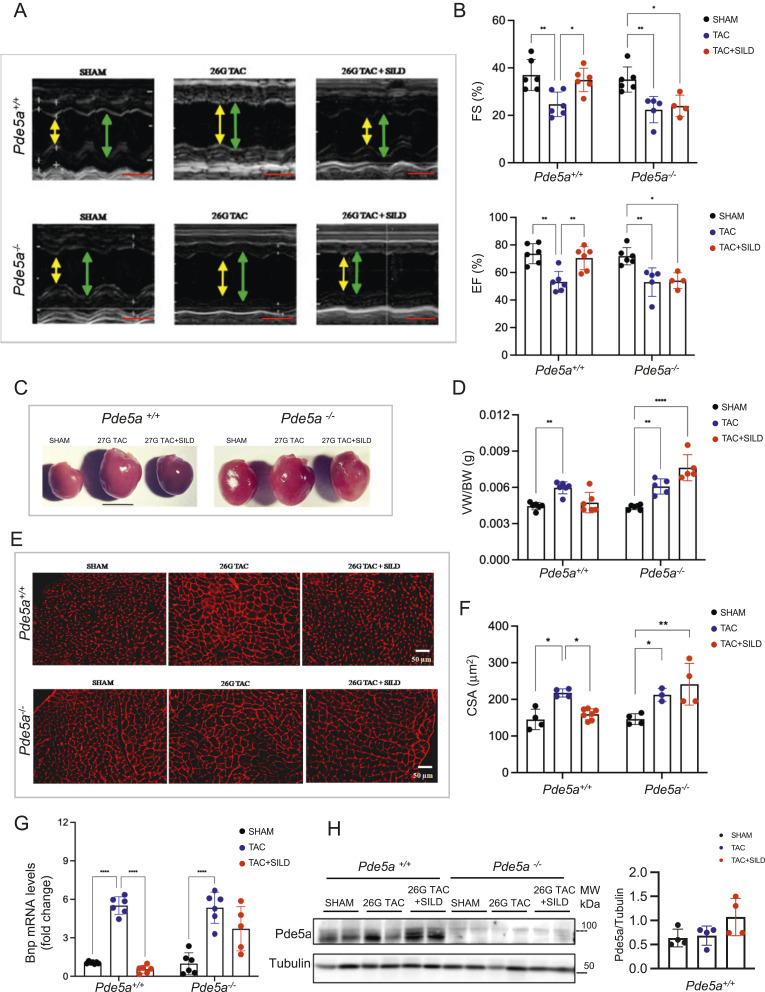
Functional, morphological, and molecular analyses of hearts of *Pde5a*^*+/+*^ and *Pde5a*^*−/−*^ mice after moderate induced hypertrophy. **(A)** Representative images of echocardiography from *Pde5a*^*+/+*^ and *Pde5a*^*−/−*^ mice under different experimental conditions. Yellow double arrows indicate left ventricular end-systolic diameter (LVESD), and green double arrows indicate left ventricular end-diastolic diameter (LVEDD). Scale bars (red) correspond to 0.1 s. **(B)** Histograms of cardiac performance for FS (%) and EF (%). **(C)** Representative pictures of freshly isolated hearts of *Pde5a*^*+/+*^ and *Pde5a*^*−/−*^ mice. Scale bar = 1 cm. **(D)** Histogram of the ratios VW/BW. **(E)** Representative pictures of transversal heart sections of *Pde5a*^*+/+*^ and *Pde5a*^*−/−*^ mice stained with wheat germ agglutinin (WGA). **(F)** Histogram of CSA of *Pde5a*^*+/+*^ and *Pde5a*^*−/−*^. **(G)** Heart mRNA expression of Bnp assessed by RT-qPCR. Fold change versus *Pde5a*^*+/+*^ SHAM is shown. **(H)** Representative picture of protein expression of Pde5a in heart samples from *Pde5a*^*+/+*^ and *Pde5a*^*−/−*^ mice. MW, molecular weight. Densitometry is shown. For each experiment, at least four mice for a group were analysed and two-way ANOVA was performed (*P ≤ 0.05, **P ≤ 0.01, ***P ≤ 0.001, and ****P ≤ 0.0001).

As expected, the analysis showed that 26G TAC induced substantial chamber dilatation and development of moderate cardiac hypertrophy with reduced left ventricular systolic function in fractional shortening (FS%) and ejection fraction (EF%) in *Pde5a*^*+/+*^ mice (Fig 2A and B). Moreover, chronic inhibition of Pde5a by Sildenafil significantly attenuated cardiac hypertrophy and cardiac dysfunction induced by 26G TAC (Fig 2A and B). Surprisingly, 26G TAC–induced cardiac hypertrophy with or without Sildenafil treatment was not prevented in *Pde5a*^*−/−*^ (Fig 2A and B). These results suggest that Sildenafil treatment but not the absence of Pde5a protects against cardiac dysfunction in mild hypertrophy.

After euthanasia, heart morphology, ventricles weight, and morphometric data were analysed in SHAM, TAC, and TAC+SILD treated mice (Fig 2C–E). Cardiac mass was measured by the ratio of ventricles and body weights (VW/BW), and an increase of heart mass after TAC was observed in both genotypes. Sildenafil treatment prevented cardiac hypertrophy only in *Pde5a*^*+/+*^mice (Fig 2C and D). Cross-sectional area (CSA) of cardiomyocytes confirmed that 26G TAC induced cell hypertrophy in both genotypes (Fig 2E and F). Sildenafil significantly reduced cardiac mass and myocyte size in *Pde5a*^*+/+*^ mice but not in *Pde5a*^*−/−*^ after 26G TAC (Fig 2E and F). The mRNA expression of brain natriuretic peptide (Bnp) was then evaluated because it is a hypertrophic marker that is quickly unregulated after increased pressure overload. The results showed that the 26G TAC model increases the Bnp mRNA expression in both genotypes. In contrast, the chronic inhibition of Pde5a with Sildenafil counteracted the increase only in the hearts of *Pde5a*^*+/+*^ mice (Fig 2G). Pde5a was not significantly modulated under the 26 TAC–induced moderate hypertrophy, whereas a slight increase was observed in the TAC+SILD group (Fig 2H).

These results suggest that the absence of Pde5a does not protect from moderate cardiac hypertrophy and the protective effect of Sildenafil inhibition requires the presence of Pde5a protein in the mouse heart.

### Cardiac fibrosis is not preserved in *Pde5a*^*−/−*^ mice after moderate TAC-induced cardiac hypertrophy, but only after Pde5a inhibition

**Figure 3.**
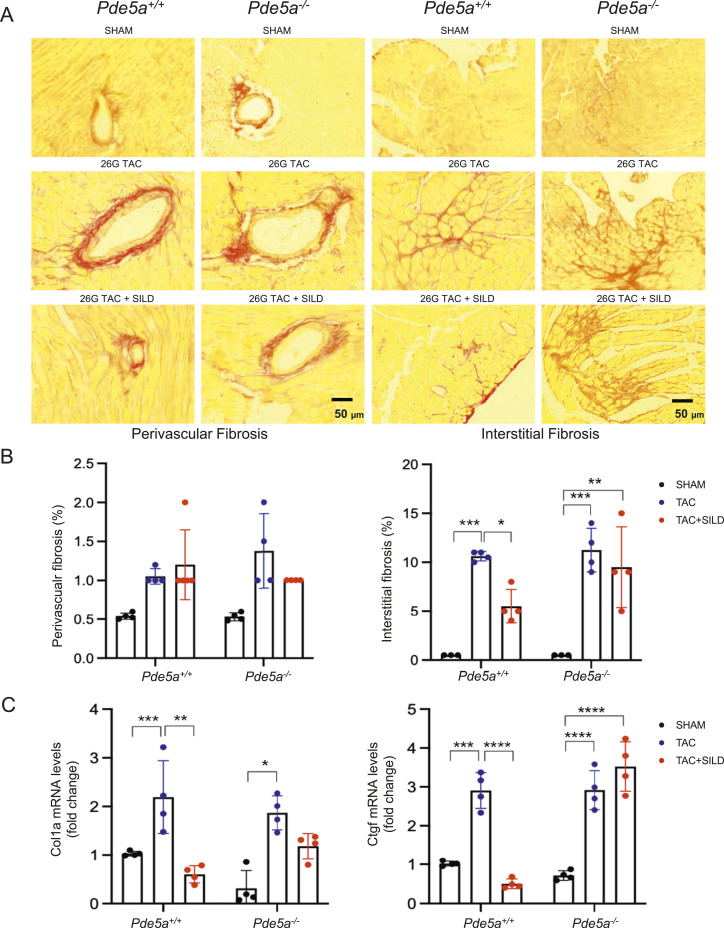
Fibrosis analysis in *Pde5a*^*+/+*^ and *Pde5a*^*−/−*^ heart sections under basal and moderate induced hypertrophy. **(A)** Representative picro sirius red (PSR) staining of transversal sections of *Pde5a*^*+/+*^ and *Pde5a*^*−/−*^ for fibrosis detection. **(B)** Histograms of interstitial and perivascular fibrosis of *Pde5a*^*+/+*^ and *Pde5a*^*−/−*^. **(C)** Heart mRNA expression of Col1a and Ctgf assessed by RT-qPCR. Fold change versus *Pde5a*^*+/+*^ SHAM is shown. For each experiment, at least three mice for a group were analysed and two-way ANOVA was performed (*P ≤ 0.05, **P ≤ 0.01, ***P ≤ 0.001, and ****P ≤ 0.0001).

To determine whether Pde5a inhibition prevents 26G TAC–induced cardiac fibrosis, heart sections were stained with picro sirius red (PSR) to detect collagen distribution in the left ventricle of the heart (Fig 3A).

As expected, 26G TAC significantly increased collagen deposition in interstitial tissues in hearts of both *Pde5a*^*+/+*^ and *Pde5a*^*−/−*^ mice (Fig 3A and B). Sildenafil reduced the percentage of interstitial collagen deposition in *Pde5a*^*+/+*^ mice but not in *Pde5a*^*−/−*^ mice after 26G TAC (Fig 3A and B). A slight increase in perivascular fibrosis was observed after TAC in both genotypes and did not change significantly after Sildenafil treatment (Fig 3A and B).

To evaluate biomarkers of fibrosis, collagen type I (Col1a) and connective tissue grow factor (Ctgf) mRNA expression were assessed by RT-qPCR. The two markers were up-regulated 2–3-folds in both mouse genotypes after 26G TAC, whereas Sildenafil reverted the fibrotic marker expression only in the hearts of injured *Pde5a*^*+/+*^ mice (Fig 3C).

These findings indicate that Pde5a absence is insufficient to prevent up-regulation of fibrosis specific markers after moderate TAC as does Sildenafil in *Pde5a*^*+/+*^ mice.

### Chronic Pde5a inhibition or Pde5a absence do not prevent cardiac dysfunction and structural alterations in severe TAC-induced cardiac hypertrophy

**Figure S1.**
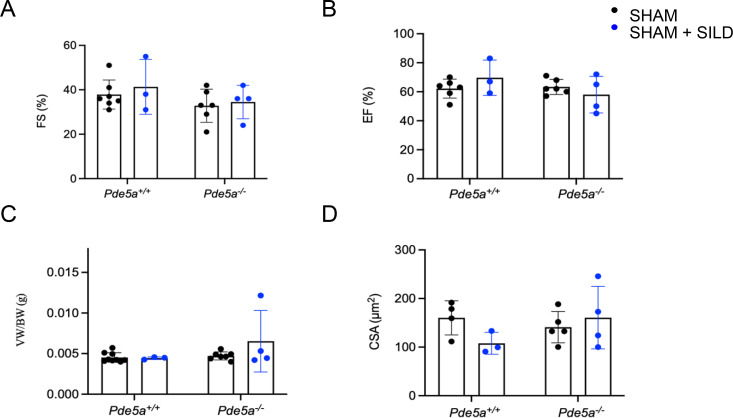
Echo and morphological parameters in hearts from *Pde5a*^*+/+*^ and *Pde5a*^*−/−*^ mice in the presence or absence of Sildenafil. **(A, B, C, D)** FS, fractional shortening; (B) EF, ejection fraction; (C) VW/BW, ventricular weight/body weight; (D) CSA, cross-sectional area. Each column represents the mean value ± SD of at least three mice.

**Figure 4.**
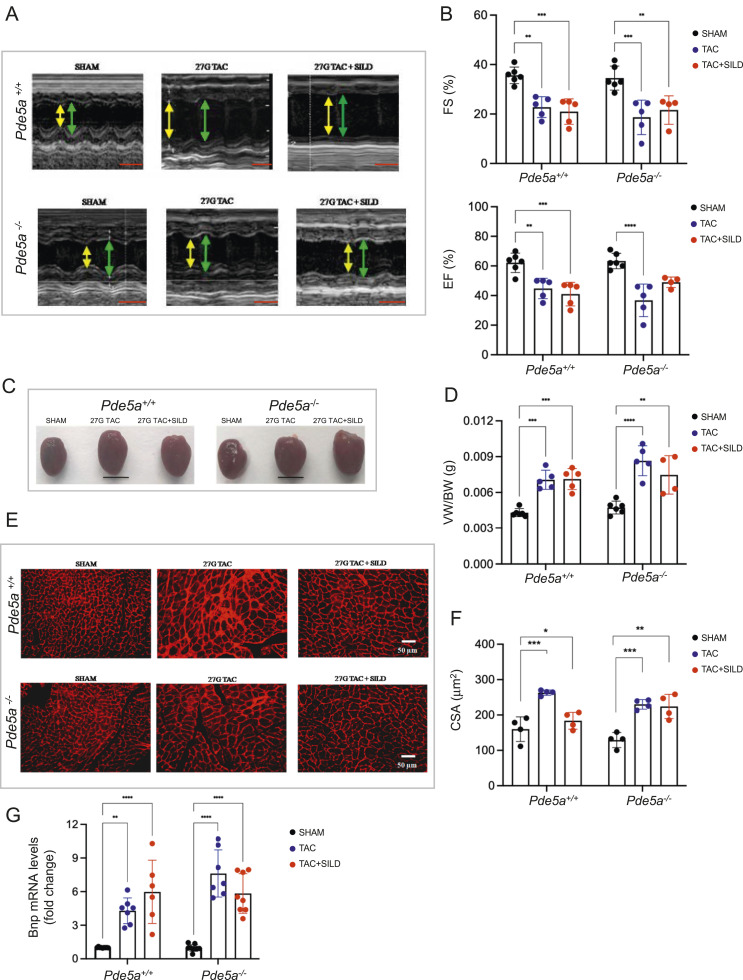
Functional, morphological, and molecular analysis of hearts of *Pde5a*^*+/+*^ and *Pde5a*^*−/−*^ mice after severe induced hypertrophy. **(A)** Representative images of echocardiography from *Pde5a*^*+/+*^ and *Pde5a*^*−/−*^ mice under different experimental conditions. Yellow double arrows indicate left ventricular end-systolic diameter (LVESD) and green double arrows indicate left ventricular end-diastolic diameter (LVEDD). Scale bars (red) correspond to 0.1 s. **(B)** Histograms of cardiac performance for FS (%) and EF (%). **(C)** Representative pictures of freshly isolated hearts of *Pde5a*^*+/+*^ and *Pde5a*^*−/−*^ mice. Scale bar = 1 cm. **(D)** Histogram of the ratios VW/BW. **(E)** Representative pictures of transversal heart sections of *Pde5a*^*+/+*^ and *Pde5a*^*−/−*^ mice stained with wheat germ agglutinin (WGA). **(F)** Histogram of CSA of *Pde5a*^*+/+*^ and *Pde5a*^*−/−*^. **(G)** Heart mRNA expression of Bnp assessed by RT-qPCR. Fold change versus *Pde5a*^*+/+*^ SHAM is shown. For each experiment, at least four mice for a group were analysed and two-way ANOVA was performed (*P ≤ 0.05, **P ≤ 0.01, and ***P ≤ 0.001).

It is known that the cardioprotective effects of Sildenafil are more evident after severe hypertrophy than those seen after moderate hypertrophy (26). Because the *Pde5a*^*−/−*^ mice are surprisingly sensitive to moderate cardiac hypertrophy and it is not counteracted by Sildenafil, a more severe cardiac hypertrophy was induced. Echocardiography revealed no major change in cardiac parameters between *Pde5a*^*+/+*^ and *Pde5a*^*−/−*^ SHAM experimental groups with or without Sildenafil administration (Table S2B and Fig S1A–D). Aortic constriction performed by 27G needles induced severe cardiac hypertrophy, and substantial left ventricular chamber dilatation was observed 4 wk after TAC in both genotypes (Fig 4A). Severe cardiac hypertrophy caused systolic dysfunction similarly to moderate hypertrophy in *Pde5a*^*+/+*^ and *Pde5a*^*−/−*^ mice (Fig 4A and B). Sildenafil treatment did not revert hypertrophy in both mouse genotypes (Fig 4A and B).

An increase in cardiac mass was observed after 27G TAC in both genotypes (Fig 4C and D). The VW/BW ratio was increased around 40% in TAC mice compared with SHAM mice, in both mouse genotypes (Fig 4D; 11% more than in 26G TAC-induced). Sildenafil treatment did not significantly prevent cardiac mass increase in both in *Pde5a*^*−/−*^ and *Pde5a*^*+/+*^ mice (Fig 4C and D). The analysis of CSA of myocytes showed that 27G TAC increased cell hypertrophy which was maintained despite Sildenafil treatment in both genotypes (Fig 4E and F). These results were confirmed by RT-qPCR for the Bnp marker (Fig 4G). The relative mRNA expression of Bnp was greater than in moderate cardiac hypertrophy and 27G TAC was able to increase Bnp mRNA expression in *Pde5a*^*+/+*^ and *Pde5a*^*−/−*^ mice, respectively.

**Figure S2.**
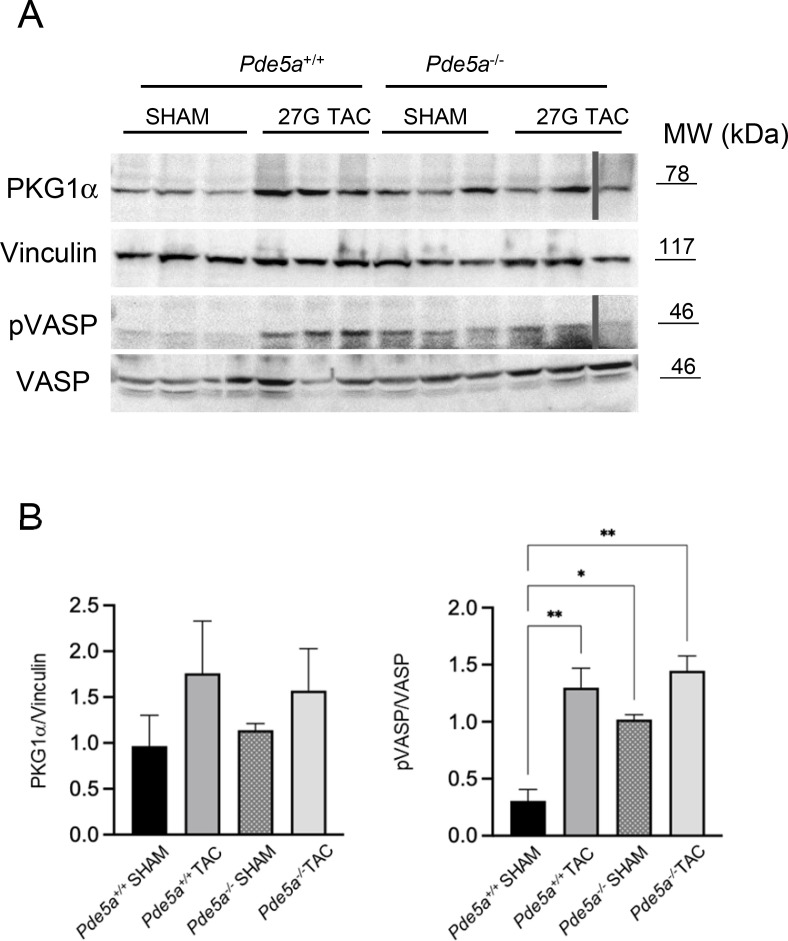
Western blot analysis of PKG signalling cascade in the heart of *Pde5a*^*+/+*^ and *Pde5a*^*−/−*^ mice. **(A, B)** Gel of protein extracts from different conditions were separated on SDS–PAGE and then probed with antibody against PKG1α, vinculin, pVASP, and VASP; (B) quantitation by densitometric analysis. One-way ANOVA was performed (*P ≤ 0.05 and **P ≤ 0.01).

Pressure overload also induces PKG activation and the phosphorylation of its specific target VASP (27). The expression level of PKG1α, the isoform more represented in heart tissue, was similar in *Pde5a*^*+/+*^ and *Pde5a*^*−/−*^ models both in physiological and after TAC conditions. pVASP was increased in *Pde5a*^*+/+*^ and *Pde5a*^*−/−*^ TAC hearts (Fig S2), indicating a similar signalling response to overload pressure. A trend of increase was observed in *Pde5a*^*−/−*^ SHAM mice.

These results suggest that the genetic ablation or pharmacological inhibition of Pde5a does not protect from severe cardiac hypertrophy.

### Fibrosis increases after severe TAC-induced cardiac hypertrophy in the hearts of *Pde5a*^*+/+*^ and *Pde5a*^*−/−*^ mice

**Figure 5.**
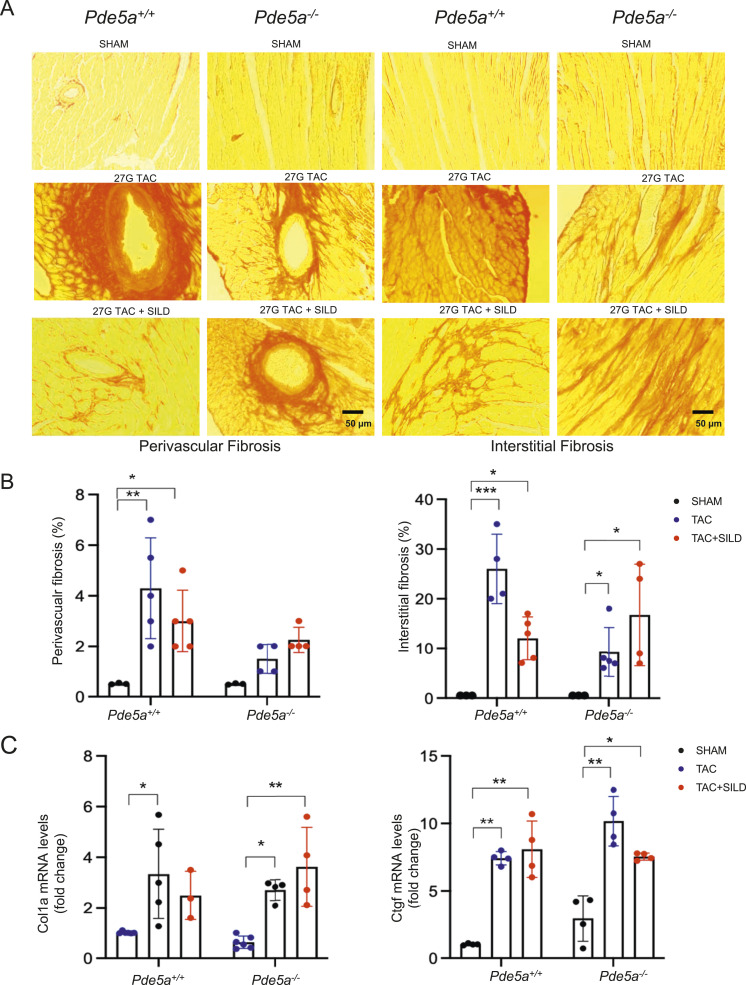
Fibrosis analysis in Pde5a^+/+^ and Pde5a^−/−^ heart sections under basal and severe induced hypertrophy. **(A)** Representative picro sirius red (PSR) of staining transversal sections of *Pde5a*^*+/+*^ and *Pde5a*^*−/−*^ for fibrosis detection. **(B)** Histograms of interstitial and perivascular fibrosis of *Pde5a*^*+/+*^ and *Pde5a*^*−/−*^. **(C)** Heart mRNA expression of Col1a and Ctgf assessed by RT-qPCR. Fold change versus *Pde5a*^*+/+*^ SHAM is shown. For each experiment, at least three mice for a group were analysed and two-way ANOVA was performed (*P ≤ 0.05 and **P ≤ 0.01).

A more pronounced presence of cardiac fibrosis was observed after 27G TAC–induced severe cardiac hypertrophy than after 26G TAC–induced moderate cardiac hypertrophy (Figs 5A–C and 3A–C). In *Pde5a*^*+/+*^ mice, an ∼25% increase in interstitial fibrosis was observed after severe hypertrophy versus 10% after moderate cardiac hypertrophy (Figs 5B and 3B). Sildenafil attenuated fibrosis in *Pde5a*^*+/+*^ heart tissues after 27 G TAC, but not in *Pde5a*^*−/−*^ mice.

After 27G TAC, Col1a mRNA expression increased around threefolds in both *Pde5a*^*+/+*^ and *Pde5a*^*−/−*^ hearts, whereas Ctgf mRNA expression increased ∼7- and 10-folds in *Pde5a*^*+/+*^ and *Pde5a*^*−/−*^ hearts, respectively (Fig 5C). Chronic inhibition of Pde5a with Sildenafil did not counteract the expression of these genes.

These findings indicate that Pde5a absence is not sufficient to prevent up-regulation of specific markers of fibrosis. Sildenafil partially counteracted cardiac fibrosis in the injured *Pde5a*^*+/+*^ hearts but not in *Pde5a*^*−/−*^ hearts.

### Cardiac Pdes expression and cyclic nucleotide levels in *Pde5a*^*+/+*^ and *Pde5a*^*−/−*^ hearts after TAC and/or Sildenafil treatment

**Figure S3.**
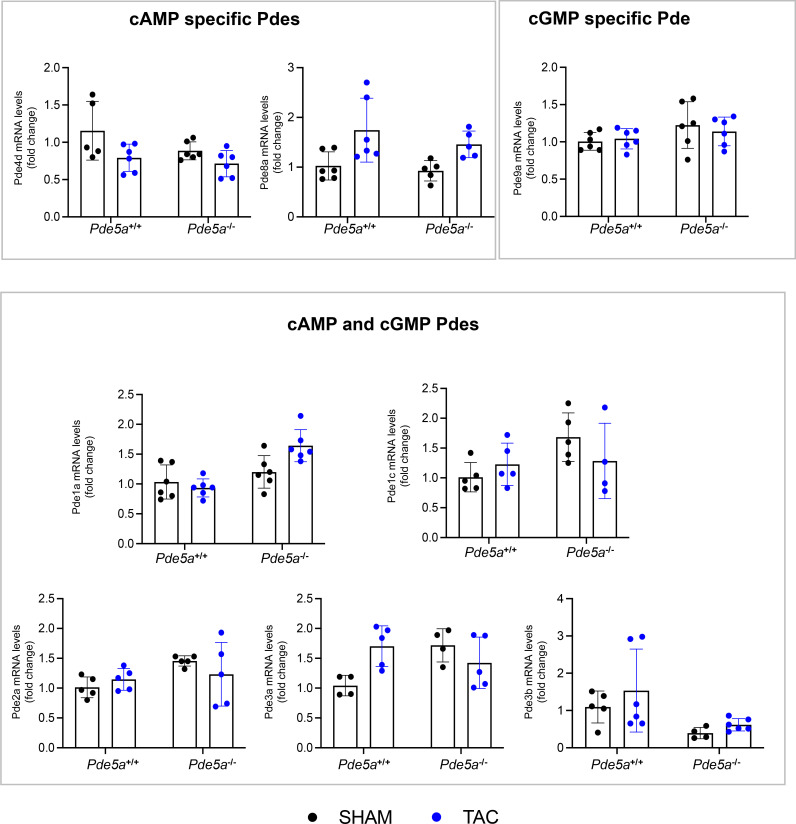
Expression of cardiac Pde isoforms in sham and TAC *Pde5a*^*+/+*^ and *Pde5a*^*−/−*^ hearts. RT-qPCR of Pde isoforms that selectively hydrolyze cAMP or cGMP or both cyclic nucleotides. Each column represents the mean value ± SD of at least four mice.

**Figure S4.**
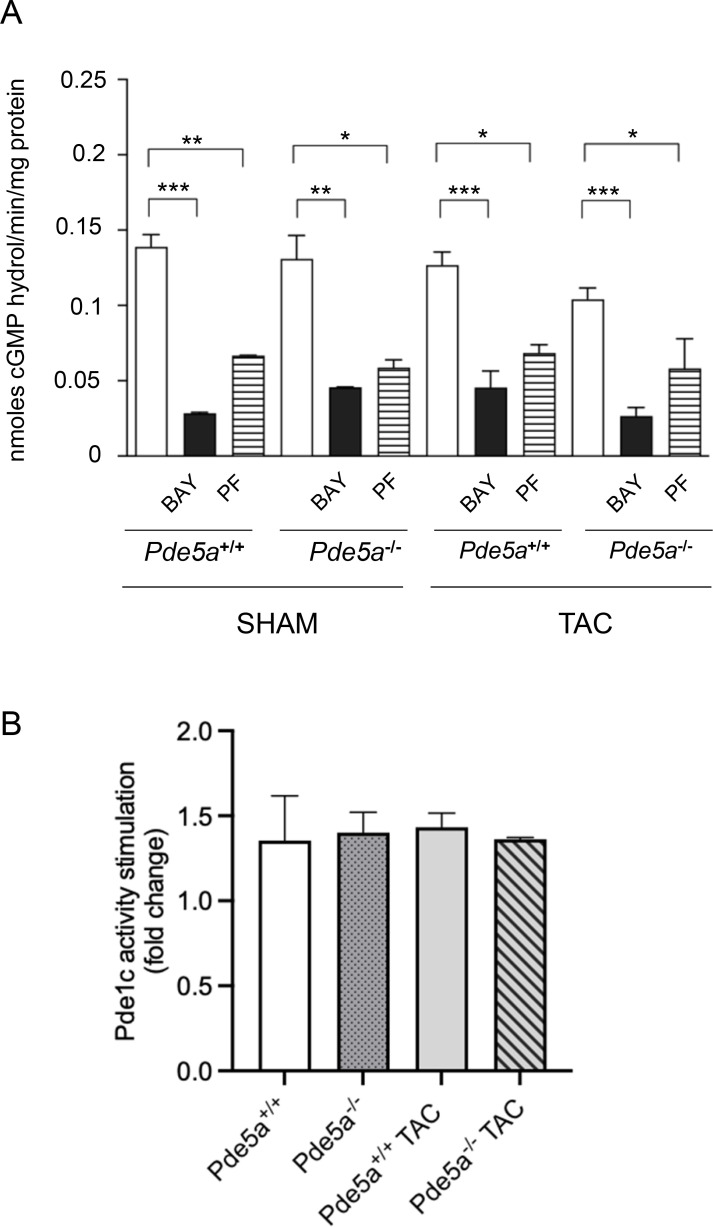
Enzymatic activity of Pde isoforms in hearts from *Pde5a*^*+/+*^ and *Pde5a*^*−/−*^ hearts. **(A)** Protein extracts were treated with the following inhibitors: Bay 60-7550 and PF04449613, specific for Pde2a and Pde9a, respectively. **(B)** Protein extracts were treated with calcium–calmodulin to specifically activate Pde1c. Each column represents the mean value ± SD of at least four mice. One-way ANOVA was performed (*P ≤ 0.05, **P ≤ 0.01, and ***P ≤ 0.001).

Because *Pde5a*^*−/−*^ mice were not protected from TAC-induced hypertrophy, transcriptional analysis was performed to test whether other cardiac-specific Pdes could compensate the Pde5a role. The results of these experiments showed no significant differences among transcripts of cardiac Pdes in *Pde5a*^*+/+*^ and *Pde5a*^*−/−*^ mice analysed under SHAM and moderate TAC (Fig S3). These data suggest that the sensitivity of *Pde5a*^*−/−*^ mice to TAC is not compensated by other Pdes at the transcriptional level. Moreover, by using selective inhibitors, we found no compensatory Pde2a and Pde9a cGMP-esterase activity in hearts from both SHAM and TAC-induced *Pde5a*^*−/−*^ mice (Fig S4A). To evaluate Pde1c activity, stimulation with calcium/calmodulin was performed because of lack of selective inhibitors and similar stimulation was observed in the two genotypes (Fig S4B).

**Figure S5.**
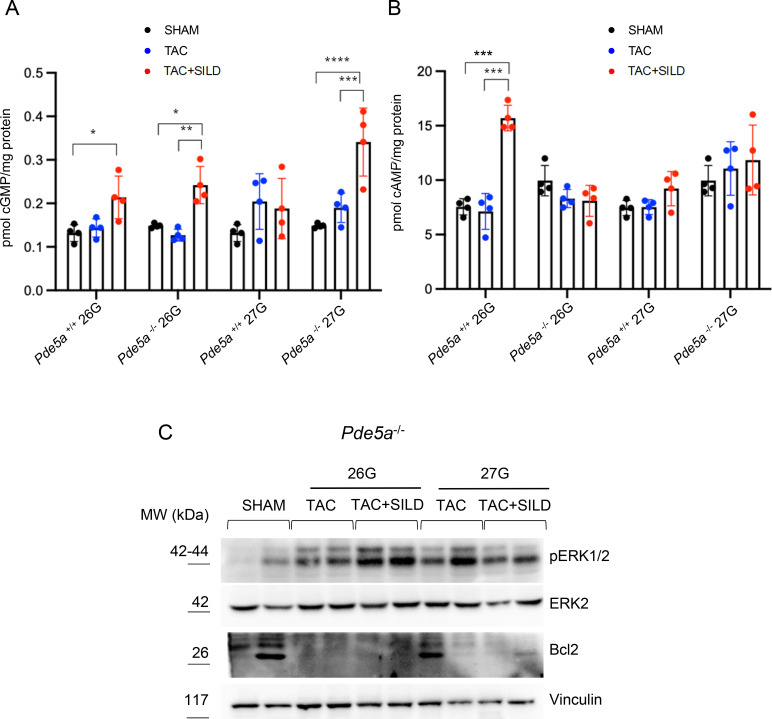
Cyclic nucleotide levels and signalling in heart samples from different experimental conditions. **(A, B)** Determination of cGMP (A) and cAMP (B) in *Pde5a*^*+/+*^ and *Pde5a*^*−/−*^ mice under SHAM, TAC, and TAC and Sildenafil conditions. Each column represents the mean value ± SD of at least four mice (two-way ANOVA, *P ≤ 0.05, **P ≤ 0.01, ***P ≤ 0.001, and ****P ≤ 0.0001). **(C)** Western blot of pERK1/2 and BCL-2 relative to ERK 2 and vinculin, respectively, in protein extracts from *Pde5a*^*−/−*^ hearts. n = 2 mice.

Furthermore, we measured cyclic nucleotide levels as a readout of Pdes activity in the presence or absence of Pde5a, under TAC experimental conditions, with or without Sildenafil. First, cGMP level was evaluated because of its known cardioprotective role. Under SHAM and TAC conditions, no major differences were observed in all groups (Fig S5A). In *Pde5a*^*+/+*^ mice, Sildenafil induced an increase in cGMP level only after moderate TAC (Fig S5A), the condition where hypertrophy was blunted (Fig 2). Unexpectedly, in *Pde5a*^*−/−*^ heart, Sildenafil triggered a great increase in cGMP level under both moderate (26G) and severe (27G) TAC conditions (Fig S5A). However, cGMP rise was unable to exert a cardioprotective role (Figs 2 and 4).

Regarding the cAMP, we observed comparable levels under SHAM and TAC conditions in both genotypes (Fig S5B), whereas the cAMP level was doubled only in 26G TAC+SILD hearts of *Pde5a*^*+/+*^ mice (Fig S5B).

**Figure 6.**
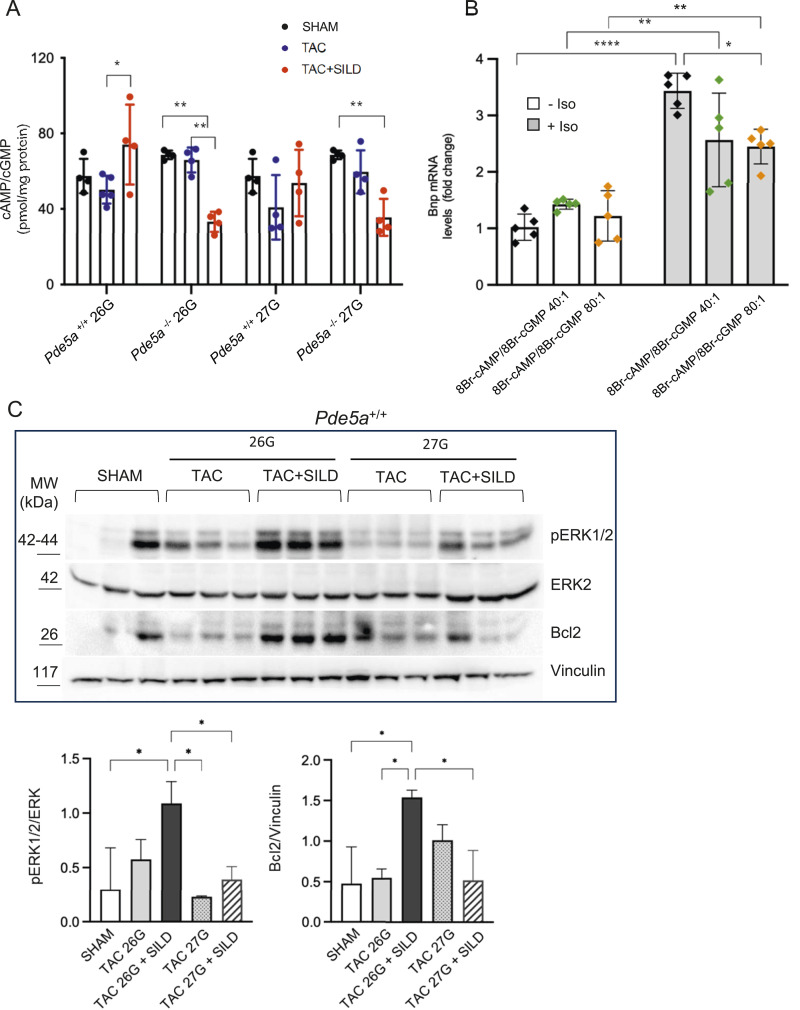
Cyclic nucleotide levels and signalling in heart samples from different experimental conditions. **(A)** Determination of cAMP/cGMP ratio in *Pde5a*^*+/+*^ and *Pde5a*^*−/−*^ mice under SHAM, TAC, and TAC and Sildenafil conditions. Each column represents the mean value ± SD of at least 4 mice (two-way ANOVA; *P ≤ 0.05 and **P ≤ 0.01). **(B)** Bnp mRNA levels in H9C2 pre-treated with isoproterenol to mimic hypertrophy and then treated with different ratio of cyclic nucleotides. Each column represents the mean value ± SD of five experiments and two-way ANOVA was performed (*P ≤ 0.05, **P ≤ 0.01, and ****P ≤ 0.0001). **(C)** Western blot and densitometry of pERK1/2 and Bcl2 relative to ERK2 and vinculin, respectively, in protein extracts from *Pde5a*^*+/+*^ hearts. Each column represents the mean value ± SD of three mice and two-way ANOVA was performed (*P ≤ 0.05).

The cAMP/cGMP ratio increases (around 80:1) only in WT hearts after 26G TAC+SILD treatment (Fig 6A), suggesting that this is the necessary condition to fulfill hypertrophy compensation. In contrast, in all the other TAC+SILD experimental groups, the cAMP/cGMP ratio is below this threshold (≤55:1), and for these reasons, the hypertrophic phenotypes could not be prevented (Fig 6A).

To confirm this result, we set up an in vitro model of cardiac hypertrophy by stimulating the cardiac cell line H9C2 with the beta-adrenergic agonist isoproterenol in different ratios of cyclic nucleotides permeable analogues (8-Br-cAMP/8-Br-cGMP 40:1 and 80:1). We measured the expression level of the hypertrophic marker BNP. Fig 6B shows that isoproterenol induces BNP mRNA after 72 h of incubation, whereas the treatment with 8-Br-cAMP and 8-Br-cGMP for the last 24 h of culture can significantly decrease the expression of BNP marker at the highest 8-Br-cAMP/8-Br-cGMP ratio. To investigate the signalling pathways underlying the effects of Sildenafil observed in *Pde5a*^*+/+*^ mice subjected to moderate versus severe pressure overload, we analysed key components of the MAPK signalling cascade, known to be modulated during cardiac hypertrophy (22, 26). Sildenafil promoted ERK1/2 phosphorylation specifically under moderate 26G TAC condition (Fig 6C). Consistent with this finding, we observed an up-regulation of the anti-apoptotic protein Bcl2 (Fig 6C), a downstream target of ERK1/2 (34), suggesting a cytoprotective effect of the drug in the context of moderate hypertrophy. In contrast, in *Pde5a* knockout mice, Sildenafil failed to increase Bcl2 expression despite a modest activation of ERK1/2 in 26G TAC (Fig S5C).

### The hearts of *Pde5a*^*−/−*^ mice undergo an anaerobic glycolytic rewiring in severe TAC-induced hypertrophy altering the LDHA/LDHB balance

Lactate dehydrogenase (LDH) has been reported to have a crucial role in adaptive cardiomyocyte growth in response to hemodynamic stress (35, 36, 37).

LDH is an omo- or hetero-tetrameric enzyme assembled from two highly similar subunits (LDHA and LDHB). The two LDH subunits can associate into five isoenzymes displaying different kinetic properties and affecting specific steps of glycolysis (37). A high amount of LDHA subunit in the hetero-tetrameric enzymes results in increased conversion of pyruvate into lactate under hypoxic conditions (37). Indeed, it was shown that TAC induces a metabolic shift towards glycolysis in WT mice, although cardiomyocytes-restricted deletion of LDHA induces heart failure (37). In contrast, under normoxic conditions, higher amounts of LDHB sustain a higher conversion of the endogenous lactate to pyruvate to feed the Krebs cycle.

**Figure 7.**
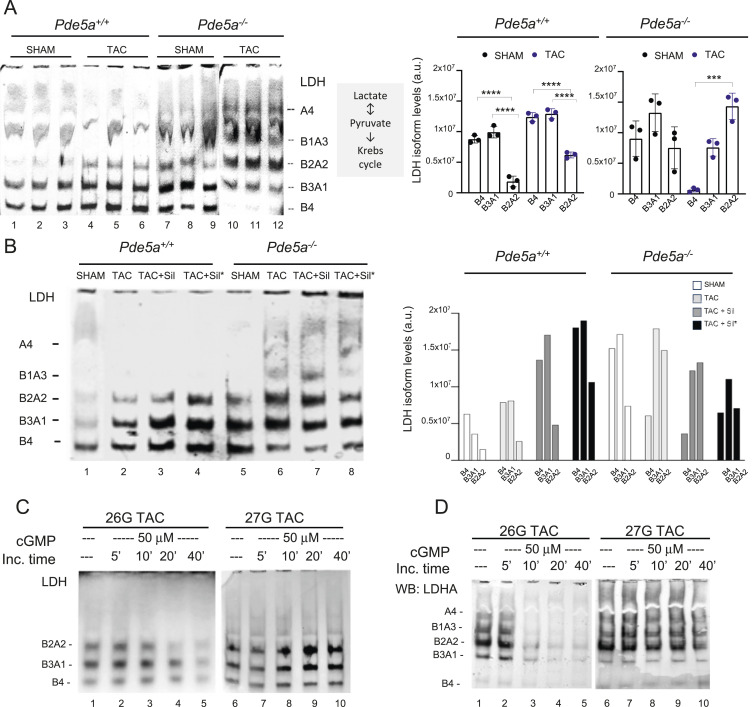
In-gel LDH-stained activities from heart protein extracts of *Pde5a*^*+/+*^ and *Pde5a*^*−/−*^ mice. **(A)** In-gel LDH staining assays with relative quantification of whole protein extracts (30 μg) from *Pde5a*^*+/+*^ and *Pde5a*^*−/−*^ (SHAM and TAC) hearts. Each column represents the mean value ± SD of at least three mice and one-way ANOVA was performed (***P ≤ 0.001 and ****P ≤ 0.0001). **(B)** Representative in-gel LDH-stained assays with relative quantification of extracts from *Pde5a*^*+/+*^ and *Pde5a*^*−/−*^ hearts (TAC and TAC+SILD conditions). **(C)** In-gel LDH staining assays of protein extracts from 26G and 27G TAC *Pde5a*^*+/+*^ hearts after in vitro addition of 50 μM cGMP for different time points. **(D)** Western blot analysis of protein extract from 26G and 27G TAC *Pde5a*^*+/+*^ hearts treated in vitro with 50 μM cGMP and probed with an antibody against LDHA C- terminal (aa 173–332).

Therefore, we checked whether, under TAC, a metabolic shift occurs in the two genotypes and to which extent. To this end, whole protein extracts from hearts of *Pde5a*^*+/+*^ and *Pde5a*^*−/−*^ mice were fractioned in native PAGE and processed to detect LDH isoforms activities by an in-gel enzymatic assay. The results, reported in Fig 7A, confirmed the involvement of LDH in the cardiac metabolic remodelling under TAC. In fact, samples obtained from *Pde5a*^*+/+*^ 27G TAC mice showed a significant increase in the B4, B3A1, and B2A2 isozymes’ activities (lanes 4–6) compared with SHAM *Pde5a*^*+/+*^ mice (lanes 1–3), suggesting an increased glycolytic flux of glucose and lactate into pyruvate, and a reduced oxidation of fatty acids.

Notably, a significant increase in the glycolytic LDHA isozyme activity (B2A2) and significant decrease in the oxidative LDHB (B4) were observed in *Pde5a*^*−/−*^ hearts from 27G TAC (lanes 10–12) versus SHAM (lanes 7–9) mice (Fig 7A).

**Figure S6.**
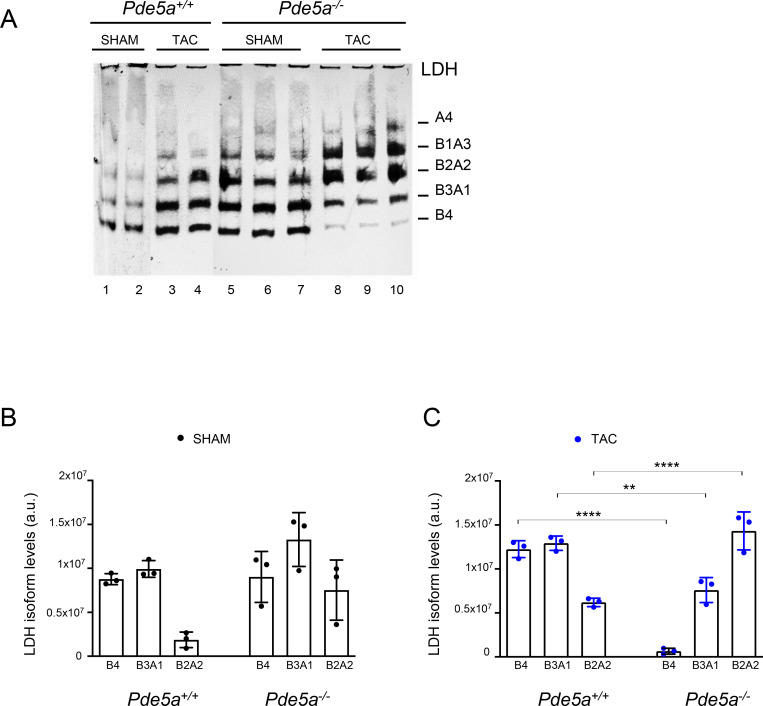
In-gel LDH-stained activities from heart protein extracts of *Pde5a*^*+/+*^ and *Pde5a*^*−/−*^ mice. **(A)** In-gel LDH staining assays of whole protein extracts (40 μg) from *Pde5a*^*+/+*^ and *Pde5a*^*−/−*^ (SHAM and 26G-TAC) hearts. **(B, C)** Relative quantification of in-gel LDH activity as shown in the main Fig 7A; statistical analysis comparing WT versus knockout mice under SHAM and TAC conditions. One-way ANOVA (**P ≤ 0.01 and ****P ≤ 0.0001).

An identical pattern of LDH activity was also observed in the 26G TAC–induced *Pde5a*^*−/−*^ hearts (Fig S6A). The LDHA/LDHB pattern in *Pde5a*^*−/−*^ SHAM and TAC is strictly directed towards lactate accumulation, when compared with SHAM and TAC of *Pde5a*^*+/+*^ hearts, respectively (Fig S6B and C).

We then tested whether Pde5a pharmacological inhibition in vivo and in vitro could modify the LDH pattern. As shown in Fig 7B, a significant increase in the oxidative LDH isozymes B4, B3A1, and B2A2 was observed in *Pde5a*^*+/+*^ 27G TAC hearts of mice treated with Sildenafil or in protein extract obtained from *Pde5a*^*+/+*^ TAC hearts with Sildenafil (lanes 3, 4 versus lane 2). On the contrary, no differences were observed in the LDH pattern of the *Pde5a*^*−/−*^ TAC samples treated with Sildenafil (lanes 7, 8 versus lane 6), in which the prominent hetero-tetrameric activities are both shifted towards the more glycolytic ones.

**Figure S7.**
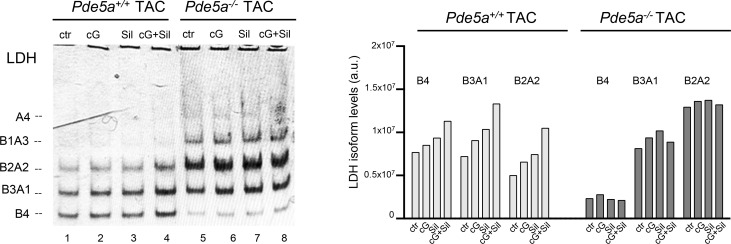
In-gel LDH-stained activities from heart protein extracts of *Pde5a*^*+/+*^ and *Pde5a*^*−/−*^ mice after TAC incubated in vitro with different drugs. In-gel LDH staining assays of protein extracts from TAC *Pde5a*^*+/+*^ and *Pde5a*^*−/−*^ hearts after in vitro addition of 50 μM cGMP (cG), 10 μM Sildenafil (Sil), or 50 μM cGMP + 10 μM Sildenafil (cG+Sil). The relative isoforms quantification was performed by densitometric analyses with Image J software and reported on the right side of each experiment.

To further confirm the role of Sildenafil in the metabolic remodelling of LDH after 27G TAC, the cGMP (cG) or Sildenafil (Sil) or cGMP plus Sildenafil (cG+Sil) was added to the same protein extract samples in vitro, incubated 40 min at 30°C, and processed for activity (Fig S7). A progressive increase in the B4, B3A1, and B2A2 activities was observed in the *Pde5a*^*+/+*^ TAC samples after incubation with these reagents, particularly when cG+Sil was added (lanes 2–4 versus lane 1). As occurred in the previous experiment, in *Pde5a*^*−/−*^ TAC samples, neither cGMP nor Sildenafil nor their combination could alter the LDH zymogram (Fig S7, lanes 6–8 versus lane 5).

**Figure S8.**
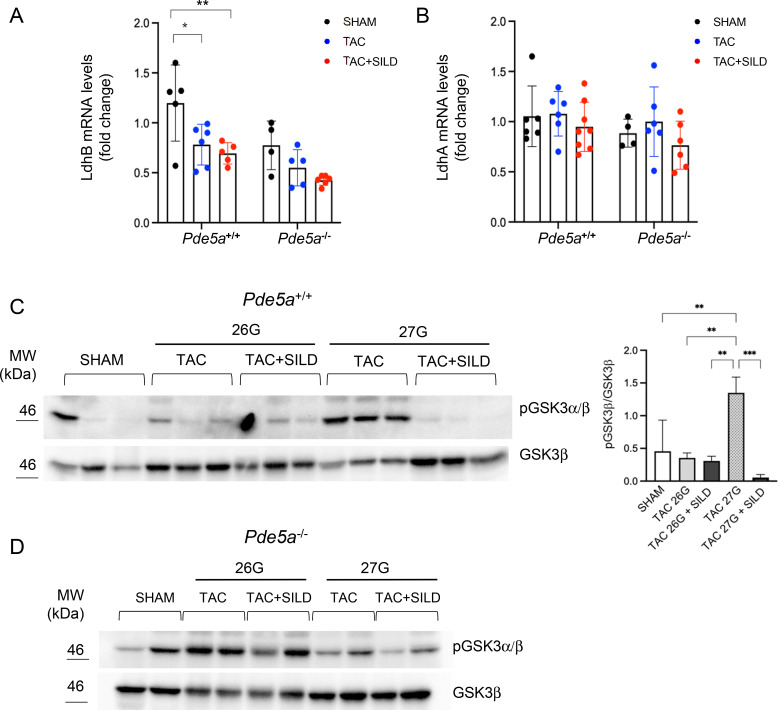
Ldh isoforms expression and metabolic signalling in *Pde5a*^*+/+*^ and *Pde5a*^*−/−*^ hearts after TAC and TAC+SILD. **(A, B)** RT–qPCR of LdhB (A) and LdhA (B) isoforms. Each column represents the mean value ± SD of at least four mice. Two-way ANOVA (*P ≤ 0.05 and **P ≤ 0.01). **(C, D)** Representative Western blot of pGSK3α/β relative to GSK3β in protein extracts from *Pde5a*^*+/+*^ (C) and *Pde5a*^*−/−*^ (D) hearts. For *Pde5a*^*+/+*^ mice, each column represents the mean value ± SD of three mice and two-way ANOVA was performed (**P ≤ 0.01 and ***P ≤ 0.001). For *Pde5a*^*−/−*^, n = 2 mice.

These data are supported by a relative decrease in LdhB mRNA levels in hearts from *Pde5a*^*−/−*^ compared with *Pde5a*^*+/+*^ mice in all conditions (Fig S8A), whereas LdhA mRNA levels are similar (Fig S8B). These data highlight critical metabolic rearrangements in hypertrophied heart of *Pde5a*^*−/−*^ mice.

Since the inhibition of Sildenafil blocks the hydrolysis of cGMP by PDE5a, to confirm the role of Sildenafil in the metabolism of hypertrophied heart, we analysed the effect of cGMP on the LDH pattern of the protein extracts from *Pde5a*^*+/+*^ 26G and 27G TAC heart mice by incubation at different time points (5-40 min). Remarkable, as shown in Fig 7C, we observed a progressive decrease in all LDH isoforms activities in the extract from the 26G TAC hearts (lanes 1–5); on the contrary, a slight increase in the LDH pattern, similarly to that shown in Figs 7B and S7, is clearly visible in 27G TAC (lanes 6–10). In a parallel experiment, an identical native PAGE gel, containing the same extracts incubated with cGMP, was transferred by Western blot to a membrane and incubated with a specific antibody against the C-terminal part of the LDHA protein. Fig 7D shows that LDHA hetero-tetramer bands progressively disappeared over time in TAC 26G (lanes 1–5), while LDH bands remained stable in TAC 27G (lanes 6–10).

Finally, we evaluated the phosphorylation of GSK3, a serine/threonine kinase with key roles in glycogen and glucose metabolism (38). Increased phosphorylation of GSK3 was observed exclusively in 27G TAC samples from WT mice (Fig S8C), suggesting enhanced glycogen accumulation and a consequent reduction in glucose availability. Notably, Sildenafil treatment prevented GSK3 phosphorylation under these conditions, indicating a possible shift towards the utilization of free glucose or lactate. In *Pde5a*^*−/−*^ mice, no major changes in GSK3 phosphorylation were detected across all conditions, despite a relatively high basal level of phosphorylated GSK3 (Fig S8D).

Together, these results clearly highlight the distinct behaviour of the 26G TAC model, in which Sildenafil is able to revert the glycolytic rewiring. In contrast, in the more severe 27G TAC model, the heart is in an irreversible critical state and fails to respond to Sildenafil, despite its molecular activity on glucose availability via the GSK3 pathway.

## Discussion

Pharmacological inhibition of Pde5a improved performance in several cardiovascular pathologies (6, 7, 10, 11, 24, 25, 39). However, the molecular mechanisms underlying the cardioprotective effect of Pde5a inhibition are still mainly unknown. To investigate this aspect and to clarify the role of Pde5a in cardiac hypertrophy induced by TAC, we used, for the first time, genetically modified mice deficient in Pde5a. The *Pde5a*^*−/−*^ mice do not show gross phenotype defects at basal condition, as other models previously generated (40, 41). The present data show that Pde5a ablation does not cause any evident phenotype alteration in the mouse body, and it does not significantly affect cardiac physiology. However, both *Pde5a*^*+/+*^ and *Pde5a*^*−/−*^ mice developed similar cardiac hypertrophy after moderate as well as after severe TAC-induced pressure overload. These results suggest the absence of Pde5a does not protect from cardiac remodelling or reduction in contractility and ejection fraction after TAC-induced moderate and severe hypertrophy. However, Sildenafil treatment, which blocks Pde5a activity, was successful in counteracting moderate hypertrophy in WT animals but did not prevent severe TAC-induced cardiac hypertrophy, differently from what was reported by references 22, 26. We cannot exclude that this discrepancy could be due to different experimental conditions (route of Sildenafil administration), choice of parameters for the inclusion in the analysis (cutoff for blood flow velocity), and time of euthanasia after surgery (4 wk). Further experiments will evaluate if a pre-treatment or a prolonged treatment with Sildenafil could also prevent hypertrophy under severe overload conditions. Except for a slight increase in cGMP after TAC and a reduction in fibrosis under moderate hypertrophy, Sildenafil did not show effects in *Pde5a*^*−/−*^ hearts, indicating its selectivity for Pde5a enzyme.

Moreover, the lack of heart protection in the absence of Pde5a was not probably due to compensatory modulation of the transcript or enzymatic activity of other Pdes. Interestingly, both cAMP and cGMP levels and their relative ratio were not modified in the hearts of both genotypes under TAC conditions, but they were modified after Sildenafil treatment of moderate hypertrophy. Sildenafil exerted cardioprotective action via cyclic nucleotides balance only when the increase in cAMP/cGMP ratio reaches a certain threshold level, as in the case of moderate hypertrophy in *Pde5a*^*+/+*^ mice. Under severe hypertrophy, an increase in cGMP was observed in the heart of *Pde5a*^*−/−*^ mice undergoing Sildenafil treatment, possibly modulating unrelated Pde5a pathways. However, this increase does not appear sufficient for cardioprotection. The different cAMP/cGMP ratio observed after Sildenafil treatment in *Pde5a*^*+/+*^ mice under the two TAC conditions, seems to correlate with increased phosphorylation of ERK1/2 kinases and up-regulation of Bcl2 as previously reported in another cardiac disease model (37). In this context, Bcl2 might counteract cardiomyocytes death in the 26G TAC+SILD condition (37).

During the chronic overload of the left ventricle, β-adrenergic receptors enhanced by Sildenafil treatment may stringently control the intracellular levels of second messengers signalling molecules (42). Because cAMP and cGMP are components of major transduction pathways controlling intracellular cardiac responses, their imbalance is responsible for the induction of cardiac hypertrophy and pathological remodelling. Indeed, we have previously shown that altered cAMP/cGMP balance occurring in another biological model system affects cell proliferation and metabolism (43). Here, we show that altered cAMP/cGMP balance is associated with cardiac metabolic rewiring in *Pde5a*^*+/+*^ and *Pde5a*^*−/−*^ mice under TAC conditions, and Sildenafil is responsible for metabolic recovery in the hearts of *Pde5a*^*+/+*^ mice.

Several reports showed that metabolic alterations are often the primary cause of cardiac failure preceding many morphological and functional changes occurring during remodelling after hypertrophy (35, 36). The development of hypertrophy by TAC triggers hypoxia, leading to reduced energy production from fatty acids towards pyruvate utilization for lactate production (37). Indeed, the glycolytic enzyme LDHA plays a significant role in adaptive cardiomyocyte growth in response to hemodynamic stress (37) and our results suggest a metabolic switch from an oxidative towards a mixed oxidative-glycolytic condition after TAC in the absence of Pde5a.

Lactate under hypoxic conditions is not only a metabolic by-product, but it is a relevant signalling molecule in many cellular systems and diseases such as heart failure, pulmonary hypertension and fibrosis, polycystic kidney disease, and cancer (35, 36, 44, 45). Sildenafil treatment in vivo or administration of cGMP and Sildenafil, in vitro, induces an increase in the expression and activity of oxidative LDHB in *Pde5a*^*+/+*^ 27G TAC mice, that, however, is not sufficient to revert cardiac hypertrophy. Conversely, in 26G TAC, the increase in cGMP level can reduce the expression and activity of the glycolytic LDHA and maintain the oxidative LDHB, thus inducing the phenotype rescue. Sildenafil does not affect the expression of oxidative LDHB in *Pde5a*^*−/−*^ TAC. We can hypothesize a specific structural role of Pde5a in maintaining the LDHA/LDHB balance that seems lost in *Pde5a*^*−/−*^ TAC mice. Because in *Pde5a*^*−/−*^ TAC mice, the chronic overload of the left ventricle leads to the shift of the LDH zymogram towards the more glycolytic LDHA activities, we speculate that Pde5a might mediate the possible role of oxygen in the regulation of LDH activities.

In line with the LDH data, during severe hypertrophy, we observed an increase in GSK3 phosphorylation, indicative of enhanced glycogen accumulation and reduced availability of glucose for oxidative metabolism. Sildenafil prevented GSK3 phosphorylation. However, this metabolic rewiring seems insufficient to achieve cardioprotection, possibly due to the lack of BCL-2 up-regulation.

It can be hypothesized that the difference between the pharmacological and genetic approach targeting Pde5a, might be due to the Sildenafil’s ability to act on cardiac metabolism through an inactivated Pde5a protein and/or to the adaptation of knockout mice, which lacks Pde5a since conception, towards normal response to pressure overload.

Further experiments are needed to clarify whether the observed metabolic rearrangement involves the interplay of Pde5a and PKG signalling and which macromolecular players are implicated. Metabolomic and translational studies will be necessary to determine whether the use of Sildenafil is beneficial to prevent/reverse hypertrophy depending on the severity of overload conditions.

**Figure 8.**
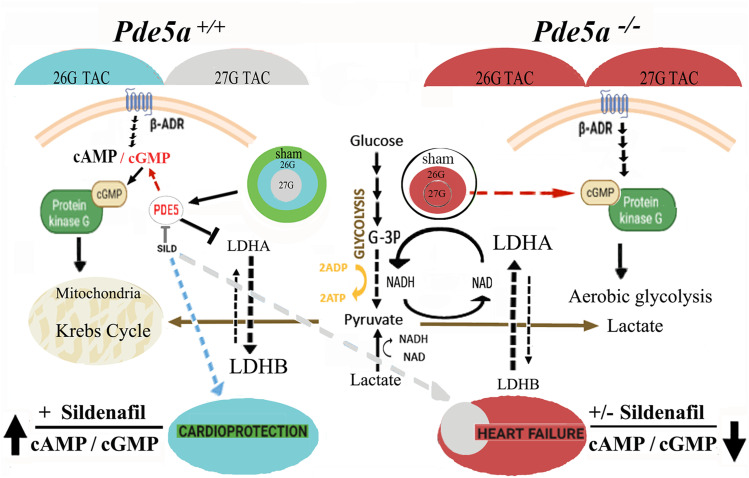
Schematic representation of signalling events occurring in cardiac hypertrophy underlining the role of Pde5a in metabolic rewiring. The circles reported in the model schematize the different degrees of aortic constriction compared with SHAM, which imposes a different metabolic regulation. In WT animals, Pde5a inhibition exerts a cardioprotective effect under moderate (blue arrow) but not severe (grey arrow) TAC-induced cardiac hypertrophy because only the former condition is effective in raising the cAMP/cGMP and LDHB/LDHA ratios. The cardiac metabolism is therefore shifted towards the oxidative mitochondrial routes and cardiac hypertrophy is prevented. Conversely, the *Pde5a*^*−/−*^ mice, always and irrespective of Sildenafil treatment, show low cAMP/cGMP and LDHB/LDHA ratios. The cardiac metabolism is therefore shifted towards aerobic glycolysis and cardiac hypertrophy progress to heart failure. In the *Pde5a*^*+/+*^ mice, the green circle indicates the sham condition, the blue and the grey circles, the 26G and 27G TAC conditions, respectively. In the *Pde5a*^*−/−*^ mice, the sham condition is indicated by white circle, whereas the 26G and 27G TAC conditions by two smaller red concentric circles. β-ADR, β-adrenergic receptor; TAC, transverse aortic constriction; G-3P, glyceraldehyde 3-phosphate; SILD, Sildenafil; NAD (H), nicotinamide adenine dinucleotide.

In conclusion, this study clarified some aspects regarding the role of Pde5a signalling in cardiac hypertrophy as illustrated in Fig 8. The first relevant result is that the lack of Pde5a does not protect from cardiac hypertrophy induction. The second point is that Pde5a inhibition can counteract the condition of mild hypertrophy at 4 wk after surgery in Pde5a WT mice by triggering a metabolic shift governed by an increased cyclic nucleotide ratio. Under this condition, an adequate oxygen perfusion should be maintained, allowing a higher percentage of the isoform LDHB and all the machinery to sustain an oxidative metabolism. Consequently, the heart can recover to normal physiology.

Under severe pressure overload, the cyclic nucleotides’ ratio is reduced and the oxidative metabolism is not preserved, making Pde5a inhibition ineffective. Lastly, the cardiac metabolic behaviour of Pde5a knockout mice under pressure overload resembles that in 27G TAC WT mice.

## Materials and Methods

### Experimental model and ethics statement

**Figure S9.**
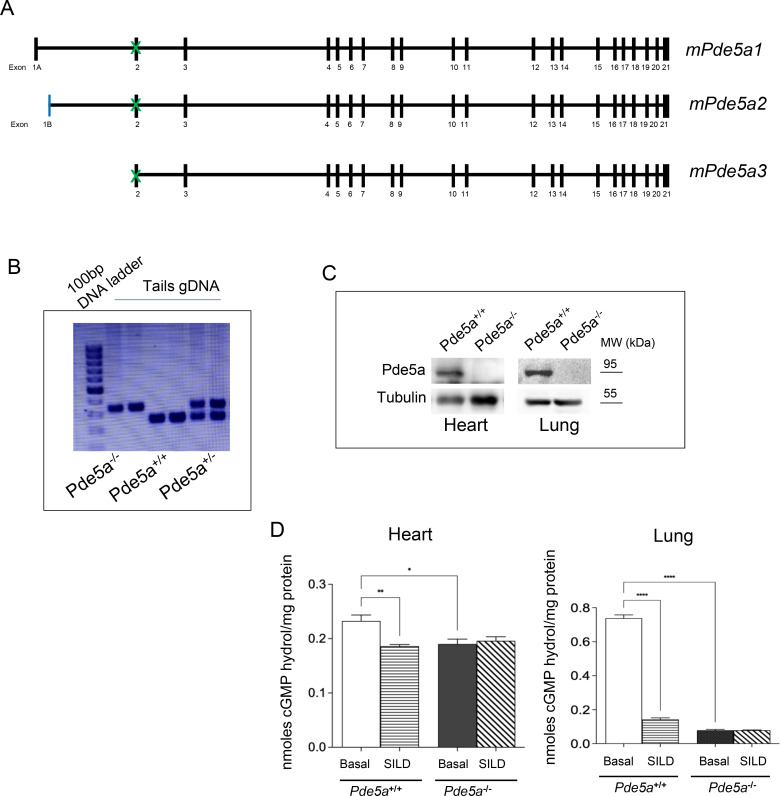
Characterization of *Pde5a*^*−/−*^ mouse model. **(A, B, C, D)** Schematic organization of the three Pde5a isoforms expressed in the heart; the green cross indicates the exon 2 deletion in the *Pde5a*^*−/−*^; (B) agarose gel showing genotype identification band of 250 bp for WT genotype and 350 bp for *Pde5a* knockout genotype; (C) Western blot analysis of Pde5a protein expression in heart and lung tissues; (D) Pde5a enzymatic activity in the absence or presence of the selective inhibitor Sildenafil in heart and lung tissues. For each experiment, four mice for a group were analysed and two-way ANOVA was performed (*P ≤ 0.05, **P ≤ 0.01, and ****P ≤ 0.0001).

*Pde5a*^*−/−*^ mice were generated at the MCGP, NCI, Frederick, MD, using Crispr/Cas9 technology to delete exon 2 of the *Pde5a* gene leading to global ablation of Pde5a protein and enzymatic activity ((13), paper submitted in Molecular Metabolism and Fig S9A–D). *Pde5a*^*−/−*^ mice were born in Mendelian ratios and had no evident physiological or behavioural abnormalities; both male and female were fertile. Similar gestational time and pups’ number were observed for *Pde5a*^*−/−*^ compared with *Pde5a*^*+/+*^ females.

Primers for genotyping mice following standard PCR protocol are as follows: forward 5′-TTG​GCA​AGG​AAT​GTG​GCT​A-3′; reverse: 5′-GCA​GGC​TTG​TTA​TTT​ACT​TAT​TTT​G-3′.

All animal experiments were performed according to the Directive 2010/63/EU of the European Parliament on the protection of animals used for scientific purposes and were conducted with the approval of the Sapienza University’s Animal Use for Research Ethics Committee and by the Italian Ministry of Health with protocol number 145/2017 PR. All the experiments were performed on *Pde5a*^*+/+*^ and *Pde5a*^*−/−*^ male mice in C57BL/6 background at 8–11 wk of age and with 25–30 g of body weight.

For chronic in vivo studies, we used Sildenafil citrate (Pfizer) 100 mg/kg/day for moderate transverse aortic constriction (TAC) and 150 mg/kg/day for severe TAC for 30 d after surgery, according to reference 22, but via gavage instead of food. Cervical dislocation was used as a method of euthanasia.

For each type of analysis, minimal group sizes were established based on the numbers needed for power calculations: G*Power 3.0.10 software using the following parameters: alfa = 0.05, 1-beta = 0.85, and d = 1. Male mice were randomly allocated into experimental groups.

### Ex vivo heart rate measurement

Ex vivo experiments were performed according to the Langendorff system (46).

Mice were anticoagulated through an intraperitoneal injection of heparin (200 μl for mice, ≥180 U/mg, H3149; Sigma-Aldrich), and 15 min later they were euthanized by cervical dislocation. Hearts were surgically removed and immediately cannulated via the aorta connected to a Langendorff apparatus and perfused with a nutrient-rich oxygenated solution, Krebs–Henseleit buffer containing 120 mM NaCl, 21 mM NaHCO_3_, 1.2 mM NaH_2_PO_4_, 5.6 mM KCl, 6 mM MgCl_2_, 2 mM CaCl_2_, 10 mM glucose, and 2 mM sodium pyruvate. Perfusion solution was gassed with a mix of 95% O_2_ + 5% CO_2_, pH 7.4, and maintained at 37°C. After 30 min of equilibration, basal heart rate was measured under constant pressure (80–100 mmHg) in basal conditions and after isoproterenol (cat. 1351005; Sigma-Aldrich) infusion.

Hearts in which the time between excision and perfusion exceeded 2 min and with heart arrhythmias were excluded from the analysis.

### H9C2 cell line culture and treatments

H9C2 cardiac cell line was purchased from ATCC and cultured in DMEM supplemented with 10% FBS (Thermo Fisher Scientific).

Cells were treated for 72 h with 10 μM isoproterenol (cat. 1351005; Sigma-Aldrich) in a medium without serum to induce cardiac hypertrophy. Cells were then treated in the presence or absence of 8-Bromo-cAMP (100 μM; cat. 203800; Sigma-Aldrich) or 8-Bromo-cGMP (2.5 μM or 1.25 μM; cat. 203820; Sigma-Aldrich) for the last 24 h. At the end of the incubation time, cells were washed, collected, and processed for further analysis.

### TAC surgery

Mice were anesthetized with an intramuscular injection of a mixture of Zoletil (tiletamine and zolazepam; Virbac) (20–40 mg/kg) and Rompun (xylazine hydrochloride; Bayer) (2.5 mg/kg), intubated, and ventilated with the mini ventilator Hugo Sachs Eletrinik Harvasr in the condition of about 150 breaths per minute and 0.3 ml of tidal volume. Partial thoracotomy to the second rib was performed under a surgical microscope, and the sternum was retracted using a chest retractor. After identification of the transverse aorta, a small piece of 7.0 silk suture was placed between the innominate artery and the left common carotid artery origins at the aortic arch. To perform TAC, two loose knots were tied around small blunt pieces of 26- or 27-gauge needles (26G, 27G) positioned parallel to the transverse aorta to generate moderate and severe cardiac hypertrophy, respectively. The chest retractor was removed, and the outflow of the mini ventilator pinched off for 2 s to reinflate the lungs. The rib cage was closed using a 5.0 silk suture with an interrupted pattern. Finally, the skin was closed using a 5.0 prolene suture with a suture pattern of isolated points. After surgery, the mice were allowed to fully recover on a warming pad and housed in standard conditions.

The conditio sine qua non to identify successful aortic arch constriction and to enroll mice in the TAC group was the value of the blood flow velocity parameter measured by Echo Doppler of aortic arch. No constriction with normal aortic peak flow velocity (usually ≤ 80 cm/s) was observed in the SHAM-operated group. Flow velocity ≥ 200 cm/s was used to enroll mice into the 26G TAC group and flow velocity ≥ 300 cm/s was used to enroll mice into the 27G TAC. Besides the flow velocity, mice were excluded from the TAC groups if two other parameters among those listed in Table S1 were not respected. Overall, around 10% of the mice, regardless of genotype, were not included in the study, possibly because of resistance to pressure overload.

In SHAM control mice, the entire procedure was identical except for the ligation of the aorta.

Survival from TAC procedure was around 95%; however, the final survival was ≥70% for moderate TAC and ≥50% for severe TAC. *Pde5a*^*−/−*^ mice showed a slightly higher than *Pde5a*^*+/+*^, but not significant, mortality after TAC (around 25% higher). Sildenafil treatment did not significantly change the mortality rate.

### Echocardiography analysis

Mice were anaesthetised by 1.5% isoflurane inhalation and an echocardiographic examination was performed by using a transthoracic, 2-D–guided M mode echocardiography equipped with an imaging transducer (HITACHI Arieta 65, FUJIFILM Europe GmbH), with a frequency range 1–18 MHz linear-array (47). Left ventricular end-systolic (LVESD) and left ventricular end-diastolic (LVEDD) internal diameters and posterior wall end-diastolic thickness were measured by the image-analysis system Metamorph, Universal Image Corporation. Percent fractional shortening was calculated as (LVEDD–LVESD/LVEDD) ×100.

### Morphological analysis

Whole hearts were fixed with 10% (vol/vol) formalin (cat. HT501128; Sigma-Aldrich) and then embedded in paraffin (Bio-Optica, Milano, Italy; cat. 087910), according to standard protocols. 7-μm sections were processed with picro sirius red (cat. 365548; Sigma-Aldrich) to identify areas of fibrosis. To analyse the cardiomyocytes’ cross-sectional area (CSA), transversal histological sections were marked with the wheat germ agglutinin (WGA; cat. 61767; Sigma-Aldrich).

The images of histological analyses were collected on a ZEISS Axioskop 2 plus microscope (Zeiss) mounting Axiocam 503 CCD camera and analysed with the ImageJ software version 1.52t; NIH.

The perivascular and interstitial fibrosis was revealed by the red of picro sirius red staining. 20X magnification of randomly selected single images of LV were analysed, in both staining. To quantify fibrosis, the heart tissue was reconstructed using ∼20 sections of each mouse in 4X magnification (ZEISS Axioskop 2 plus microscope mounting Axiocam 503 CCD camera and Adobe Photoshop 5.0). The images were analysed using the ImageJ software (version 1.52t; NIH).

### RNA isolation and analysis

Total RNA was isolated from tissue samples using the RNA purification kit from Zymo Research (cat. R2050) according to the manufacture’s protocol. The yield and purity of RNA was determined with the NanoDrop OneC microvolume UV-Vis Spectrophotometer (Thermo Fisher Scientific). RNA was reverse transcribed by Maxima H Minus Reverse Transcriptase (cat. EP0751; Thermo Fisher Scientific) according to the manufacturer’s protocol and then processed for quantitative PCR (qPCR). qPCR reaction was carried out by using PowerUp SYBR green Master Mix (cat. A25743; Thermo Fisher Scientific). Target transcripts were analysed using QuantStudio 7 Flex RT–PCR System (Thermo Fisher Scientific). For the quantification analysis, the comparative threshold cycle (Ct) method was used. The comparative threshold cycle (Ct) method was used for the quantification analysis. The Ct values of each gene were normalized to the Ct value of HPRT in the same RNA sample. The gene expression levels were evaluated by fold change using the equation 2^−ddCt^. Samples are expressed as the fold change compared with WT mice samples. All values represent the mean ± SD of at least four different RNA preparations. The primers used in qPCR assays are reported in Table S3; their efficiency was calculated using the serial dilution method.

Table S3. Sequence of primers used for RT-qPCR.

### Protein analysis

Heart tissues were lyzed with RIPA lysis buffer (cat. 20-188; Sigma-Aldrich) with the addition of protease inhibitor cocktail (cat. 11836153001; Sigma-Aldrich), 1 mM dithiothreitol (cat. DTT-RO; Sigma-Aldrich), 1 mM β-glycerophosphate (cat. G9422; Sigma-Aldrich), and 0.5 mM sodium orthovanadate (cat. S6508; Sigma-Aldrich) and homogenized using the TissueLyser sample disrupter (QIAGEN). The protein concentration was determined by Bradford assay (Serva Electrophoresis GmbH). Aliquots of equal amounts (50 μg) of proteins added with Laemmli loading buffer were separated on 10% or 12% sodium dodecyl sulfate polyacrylamide gel electrophoresis (SDS–PAGE). For LDHA isoforms protein expression, heart tissues were loaded in native gels as described below.

Proteins were then transferred to Immobilon PVDF membrane (cat. IPVH00010; Sigma-Aldrich), blocked for 1 h at RT in 5% milk in TBST-T (150 mM NaCl, 50 mM Tris–HCl, 0.1% Tween 20, pH 7.6; cat. P9416; Sigma-Aldrich). Membranes were probed with specific primary antibodies (anti-Pde5a, cat. 2395; Cell Signaling; anti-Tubulin, cat. T5168; Sigma-Aldrich; anti-vinculin, cat. V9264; Sigma-Aldrich; anti-PKG1α, cat. 13511; Cell Signaling Technology; anti-VASP, cat Sc-13975; Santa Cruz; anti-pVASP, cat. 3114; Cell Signaling Technology, anti-LDHA, cat. Sc-133123; SantaCruz Biotechnology; anti-GSK3β, cat. 9315S; Cell Signaling; anti-pGSK3α (Ser21)/β (Ser9), cat. 9331; Cell Signaling Technology; anti-ERK2, cat. sc-154; SantaCruz Biotechnology; anti-pERK1/2, cat. sc-7583; SantaCruz Biotechnology; cat. sc-7985; gy; anti BCL-2, cat. sc-783; SantaCruz Biotechnology) diluted in 5% BSA/TBS-T or 5% milk/TBS-T solution and incubated overnight at 4°C. After washing and incubation with secondary antibodies, the membranes were washed and the signals were detected by chemiluminescence with the ECL SuperSignal West Pico PLUS substrate (cat. 3450; Thermo Fisher Scientific). Chemiluminescent images of immunodetected bands were recorded with the Syngene G-box system (Syngene Bioimaging), and immunoblot intensities were quantitatively analysed using ImageJ software (NIH). The results representing the mean of at least three independent experiments were normalized to the amount of housekeeping proteins.

### LDH activity

LDH activity was assayed by an in-gel enzymatic assay on heart extracts separated on native polyacrylamide gel electrophoresis (PAGE). Native PAGE was performed with 5% non-denaturing acrylamide gel with a Tris/glycine pH 8.8 running buffer at 4°C for 60–70 min under a current of 20 mA in a Bio-Rad Mini-Protean electrophoresis apparatus (Bio-Rad) (43, 48, 49).

Each well was loaded with 30–50 μg of total protein extracts from heart tissues in RIPA buffer. In in vitro experiments, protein samples were pre-incubated at 30°C for 40 min with Sildenafil (10 μM) or cGMP (50 μM; cat. G6129; Sigma-Aldrich) or both to determine the effects of these reagents on the LDH activities’ balance. After electrophoresis, the gel was stained in 5 ml of the following solution containing 25 μl NAD^+^ (stock solution 100 mg/ml in 100 mM Tris–HCl, pH 8; cat. N0632; Sigma-Aldrich), 20 μl nitrotetrazolium blue chloride (NTB, stock solution 40 mg/ml in H_2_O; cat. N6876; Sigma-Aldrich), 10 μl phenazine methosulfate (PMS, stock solution 40 mg/ml in H_2_O; cat. P9625; Sigma-Aldrich), 30 μl D-L lactate 40% solution (cat. L4263; Sigma-Aldrich), and water up to 5 ml.

Gels were stained at RT in the dark until bands became visible (roughly 10 min). The isoforms were identified by their different gel mobility and relatively quantified by densitometric analysis with the ImageJ software (version 1.52t; NIH).

### Cyclic nucleotide assay

cAMP and cGMP were measured with a Direct cAMP/cGMP ELISA Kit (cat. ADI-900-066; ADI-900-014; Enzo Life Sciences) according to the manufacturer’s instruction. Heart tissues were homogenized in 0.1 M HCl with 0.1% vol/vol Triton X-100 with glass beads in a TissueLyser sample disrupter (QIAGEN). Lysates were then centrifuged at 14,000*g* for 10 min. Aliquots of 100 μl were analysed to determine nucleotide levels following the acetylated version of the assay, according to the kit instructions.

Aliquots of 5 μl of the supernatant were neutralized and assayed for protein determination with Bradford assay. Results are referred to cAMP and cGMP standard curves performed together with the cAMP and cGMP assay. Cyclic nucleotide levels were normalized to the protein content of each sample. Values represent the mean ± SD of at least four sample preparations.

### Pdes activity assay

Tissues were homogenized in a 20 mM Tris–HCl buffer, pH 7.2, with 0.2 mM EGTA, 5 mM MgCl_2_, 1 mM phenylmethylsulphonyl fluoride, 5 mM 2-mercaptoethanol, 2% (vol/vol) antiprotease cocktail (cat. P8340; Sigma-Aldrich), and 0.1% Triton X-100 using a mini-glass homogenizer (15 strokes, 4°C). All procedures were performed at 4°C. The homogenate was centrifuged at 14,000*g* for 30 min at 4°C and the resulting supernatants were used for further analysis.

Pde activity was measured with the method described by Thompson and Appleman (50) at 30°C in 60 mM Hepes, pH 7.2, 0.1 mM EGTA, 5 mM MgCl_2_, 0.5 mg/ml BSA, and 30 mg/ml soybean trypsin inhibitor, in a final volume of 0.15 ml. The reaction was started by adding tritiated substrates at a final concentration of 1 μM [^3^H] cGMP and stopped by adding 0.1 N HCl. After neutralization with 0.1 N NaOH in 0.1 M Tris–HCl, pH 8.0, 2 mg/ml 5′-nucleotidase (snake venom from *Crotalus atrox*; Sigma-Aldrich) in 0.1 M Tris–HCl, pH 8.0, was added. Samples were gently mixed and incubated for 30 min to allow complete conversion of 5′-nucleotide to its corresponding nucleoside. Unhydrolyzed cyclic nucleotide and the corresponding nucleoside were separated by DEAE-Sephadex A-25 columns. The eluate was mixed with ULTIMA GOLD scintillation liquid (PerkinElmer Inc.) and counted on a Tri-Carb 2100TR Liquid Scintillation Counter (2000CA; Packard Instruments). To evaluate the enzymatic specific activity of each Pdes, the specific inhibitors were added to the reaction mix at the following concentration: 0.1 μM Sildenafil (Pde5a inhibitor; Pfizer), 0.1 μM BAY 60-7550 (Pde2 inhibitor, cat. SML2311; Sigma-Aldrich), and 0.3 μM PF04449613 (Pde9 inhibitor, cat. 5915; TOCRIS).

### Statistical analysis

Statistical analysis was performed with GraphPad Prism software (GraphPad). All data are expressed as mean ± SD and analysed with one- and two-way ANOVA with Tukey’s correction (Tukey’s post hoc test). The differences were considered significant if *P ≤ 0.05, **P ≤ 0.01, ***P ≤ 0.001, and ****P ≤ 0.0001.

## Supplementary Material

Reviewer comments
