# Supplementary material for: The rewiring of cAMP/cGMP and LDH signalling drives cardiac hypertrophy in Pde5a−/− mice
Source: Life Sci Alliance. 2025 Jul 14;8(10):e202403094. doi: 10.26508/lsa.202403094 (PMC12261138; doi:10.26508/lsa.202403094)
Supplement: Supplementary file 1 [file LSA-2024-03094_TableS1.docx]

**Supplementary Table 1: Cut-off of parameters used to select mice with heart hypertrophy**

| **Heart** | **VW/BW (g)** | **CSA (µm^2^)** | **FS (%)** | **EF (%)** | **Blood flow**  **velocity (cm/s)** |
| --- | --- | --- | --- | --- | --- |
| Hypertrophy | > 0.005 | > 180 | < 30 | < 60 | 200-300 (26G)  >300 (27G |

VW/BW ventricle weight/body weight; CSA cross sectional area; FS fractional shortening; EF ejection fraction. Blood flow velocity was measured at the constriction site to enroll mice in the TAC groups (see Huang et al., Front. Phsiol.; [doi: 10.3389/fphys.2022.1026884](file:///D:\#doi: 10.3389/fphys.2022.1026884)).
