# Supplementary material for: The rewiring of cAMP/cGMP and LDH signalling drives cardiac hypertrophy in Pde5a−/− mice
Source: Life Sci Alliance. 2025 Jul 14;8(10):e202403094. doi: 10.26508/lsa.202403094 (PMC12261138; doi:10.26508/lsa.202403094)
Supplement: Supplementary file 2 [file LSA-2024-03094_TableS2.docx]

**Supplementary Table 2A: Echo parameters**

| **ECHO**  **Parameters** | ***Pde5a^+/+^***  **SHAM** | ***Pde5a^+/+^***  **26G TAC** | ***Pde5a^+/+^***  **26G TAC+SILD** |
| --- | --- | --- | --- |
| ***n*** | 6 | 6 | 6 |
| **HR (bpm) ±SD** | 413±63 | 414±71 | 359±70 |
| **LVEDD (mm) ±SD** | 3.75±0.50 | 4.18±0.35 | 3.90±0.47 |
| **LVESD (mm) ±SD** | 2.45±0.55 | 3.02±0.56 | 2.62±0.44 |
| **LVWTD (mm) ±SD** | 0.72±0.12 | 0.72±0.17 | 0.72±0.23 |
| **LVWTS (mm) ±SD** | 1.17±0.24 | 1.00±0.30 | 1.07±0.23 |
| **Aorta (mm) +SD** | 1.23±0.14 | 1.33±0.14 | 1.33±0.16 |
| **ECHO**  **Parameters** | ***Pde5a^-/-^***  **SHAM** | ***Pde5a^-/-^***  **26G TAC** | ***Pde5a^-/-^***  **26G TAC+SILD** |
| ***n*** | 6 | 5 | 5 |
| **HR (bpm) ±SD** | 356±24 | 393±63 | 394±61 |
| **LVEDD (mm) ±SD** | 3.92±0.37 | 4.55±0.50 | 3.95±0.98 |
| **LVESD (mm) ±SD** | 2.56±0.39 | 3.55±0.63 | 2.90±0.80 |
| **LVWTD (mm) ±SD** | 0.7±0.15 | 0.85±0.09 | 0.63±0.11 |
| **LVWTS (mm) ±SD** | 1.04±0.29 | 1.22±0.18 | 1.05±0.11 |
| **Aorta (mm) +SD** | 1.32±0.17 | 1.43±0.13 | 1.33±0.18 |

Data shown in Mean ± Standard Deviation (SD). ***n****:* number of mice; **HR**: Heart Rate; **LVEDD** and **LVESD**: Left Ventricle End-Dimension at Diastole and Systole; **LVWTD** and **LVWTS**: Left Ventricle Wall Thickness at Diastole and Systole

**Supplementary Table 2B: Echo parameters**

| **ECHO**  **Parameters** | ***Pde5a^+/+^***  **SHAM** | ***Pde5a^+/+^***  ***SILD*** | ***Pde5a^+/+^***  **27G TAC** | ***Pde5a^+/+^***  **27G TAC+SILD** |
| --- | --- | --- | --- | --- |
| ***n*** | 6 | 4 | 5 | 5 |
| **HR (bpm) ±SD** | 432±55 | 441+53 | 466±33 | 395±67 |
| **LVEDD (mm) ±SD** | 3.75±0.24 | 3.6+0.54 | 4.06±0.26 | 4.04±0.15 |
| **LVESD (mm) ±SD** | 2.42±0.25 | 2.17+0.66 | 3.04±0.26 | 3.06±0.24 |
| **LVWTD (mm) ±SD** | 0.70±0.24 | 0.50+0.01 | 0.66±0.08 | 0.58±0.13 |
| **LVWTS (mm) ±SD** | 0.93±0.33 | 1.03+0.09 | 0.98±0.10 | 0.96±0.16 |
| **Aorta (mm) +SD** | 1.15±0.14 | 1.20+0.08 | 1.32±0.18 | 1.36±0.13 |
| **ECHO**  **Parameters** | ***Pde5a^-/-^***  **SHAM** | ***Pde5a^-/-^***  **SILD** | ***Pde5a^-/-^***  **27G TAC** | ***Pde5a^-/-^***  **27G TAC+SILD** |
| ***n*** | 6 | 4 | 5 | 4 |
| **HR (bpm) ±SD** | 395±54 | 359+11 | 438±68 | 442±12 |
| **LVEDD (mm) ±SD** | 4.20±0.33 | 4.03+0.15 | 4.88±0.55 | 4.17±0.30 |
| **LVESD (mm) ±SD** | 2.83±0.46 | 2.67+0.47 | 3.98±0.70 | 3.22±0.26 |
| **LVWTD (mm) ±SD** | 0.58±0.13 | 0.77+0.21 | 0.76±0.27 | 0.82±0.15 |
| **LVWTS (mm) ±SD** | 1.07±0.27 | 1.30+0.30 | 1.04±0.35 | 1.32±0.22 |
| **Aorta (mm) +SD** | 1.23±0.11 | 1.30+0.10 | 1.32±0.15 | 1.48±0.19 |

Data shown in Mean ± Standard Deviation (SD). ***n****:* number of mice; **HR**: Heart Rate; **LVEDD** and **LVESD**: Left Ventricle End-Dimension at Diastole and Systole; **LVWTD** and **LVWTS**: Left Ventricle Wall Thickness at Diastole and Systole
