# Supplementary material for: The rewiring of cAMP/cGMP and LDH signalling drives cardiac hypertrophy in Pde5a−/− mice
Source: Life Sci Alliance. 2025 Jul 14;8(10):e202403094. doi: 10.26508/lsa.202403094 (PMC12261138; doi:10.26508/lsa.202403094)
Supplement: Supplementary file 3 [file LSA-2024-03094_TableS3.docx]

**Supplementary Table 3: sequence of primers utilized for RT-qPCR**

| **Gene** | **Forward primer** | **Reverse primer** |
| --- | --- | --- |
| mBnp  rBNP | 5’-AAGTCCTAGCCAGTCTCCAG-3’  5’-GTCAGTCGCTTGGGTGT-3’ | 5’-GAGCTGTCTCTGGGCCATTT-3’  5’-CCAGAGCTGGGAAAGAAG-3’ |
| Col1a | 5-’TAAAGGTGCCAATGGTGCTC-3’ | 5’-CCAGGTTCACCACTGTTACC-3’ |
| Ctgf | 5’-TGCGAAGCTGACCTGGAGGA-3’ | 5’-GAGCGGTCGTTGTACAGAAT-3’ |
| Hprt | 5’-GACTTGCTCGAGATGTCATG-3’ | 5’-TGTAATCCAGCAGGTCAGCA-3’ |
| LdhA | 5’-CCGTTACCTGATGGGAGAGA-3’ | 5’-TGCCCAGTTCTGGGTTAAGA-3’ |
| LdhB | 5’-CTCAGATCGTCAAGTACAGCC-3’ | 5’-ATCCGCTTCCAATCACACGGTG-3’ |
| Pde1A | 5’-AGGTATCATGCACTGGCTCA-3’ | 5’-GAGCGGTCGTTGTACAGAAT-3’ |
| Pde1C | 5’-ATGGGGATGATGCTTAGGAG-3’ | 5’-CAATGCTTCGATTACAGCCG -3’ |
| Pde2A | 5’-ACCGAAAGATCCTGCAACTG-3’ | 5’-TTCTCCCAGCACTTTGTCTC-3’ |
| Pde3A | 5’-AGAATCCATGCCACCGATGT-3’ | 5’-CCCATGTGTCCGTGTGTAAA-3’ |
| Pde3B | 5’-ATGGGTGCTTTCTCAGGTTC-3’ | 5’-TTGGAGTTTTGCTAGTTGAGGA-3’ |
| Pde4D | 5’-GCCTCTGACTGTTATCATGCAC-3’ | 5’-GAGTTGGGAAACTGGTTCTG-3’ |
| Pde8A | 5’-TCAGAGTGTGCAATGGCAAC-3’ | 5’-GTCCATCGAATGTTTCCTCC-3’ |
| Pde9A | 5’-CTACGAGGAGCTGAAGCAGC-3’ | 5’-TGTAATCCAGCAGGTCAGCA-3’ |
